# EMBalance - validation of a decision support system in the early diagnostic evaluation and management plan formulation of balance disorders in primary care: study protocol of a feasibility randomised controlled trial

**DOI:** 10.1186/s13063-016-1568-x

**Published:** 2016-09-05

**Authors:** Laura Rammazzo, Dimitris Kikidis, Amal Anwer, Nora Macdonald, Efthymios Kyrodimos, Christoph Maurer, Floris Wuyts, Linda Luxon, Athanasios Bibas, Doris-Eva Bamiou

**Affiliations:** 1 Neuro-otology Department, National Hospital for Neurology and Neurosurgery, Queen Square, London, WC1N 3BG UK; 2 UCL Ear Institute, University College London, 332 Gray’s Inn Road, London, WC1X 8EE UK; 3 First Department of Otolaryngology-Head and Neck Surgery, ‘Hippokrateio’ General Hospital, University of Athens, Vassilissis Sophias Avenue 114, 115 27 Athens, Greece; 4 Department of Neurology, University of Freiburg, Hugstetter Straße 55, 79106 Freiburg, Germany; 5 Research Centre for Equilibrium and Aerospace (AUREA), Antwerp University, Wilrijkstraat 10 Route 71, B904, Edegem, 2650 Antwerp Belgium; 6 Royal College of Physicians, 11 St. Andrews Place, Regent’s Park, London, NW1 4LE UK

**Keywords:** Vestibular disease, Dizziness, Vertigo, Decision support system, Randomised controlled trial, Feasibility study

## Abstract

**Background:**

Balance problems are caused by multiple factors and often lead to falls and related fractures, bringing large socio-economic costs. The complexity of balance control mechanisms, the lack of medical expertise, and the absence of specialised equipment contribute to the delayed or incorrect diagnosis and management ofthese patients. Advances in computer science have allowed the development of computer systems that support clinical diagnosis and treatment decisions based on individualised patient data. The aim of the EMBalance decision support system (DSS) is to support doctors facing this clinical challenge, to make a definitive diagnosis and implement an effective management plan. The EMBalance study will determine the accuracy of this supportive tool when used by non-specialist doctors. This study is funded by the European Union’s Seventh Framework Programme.

**Methods/design:**

EMBalance is a proof-of-concept study designed as a non-commercial, international, multi-centre, single-blind, parallel-group randomised controlled trial to be carried out at four clinical sites in the United Kingdom, Germany, Greece and Belgium. The study is comprised of three stages: internal pilot, phase I (diagnosis) and stage II (management). For this purpose, 200 patients presenting with persistent dizziness (>3 months’ duration) to primary care services will be randomised to either the intervention group (diagnostic assessment with the DSS) or a control group (diagnostic assessment without the DSS). Patients allocated to the intervention group will be assessed by a doctor with the support of the EMBalance DSS, while patients allocated to the control group will receive a visit as per standard practice. Ultimately, all patients’ diagnoses and management plans will be certified by a consultant in neuro-otology.

**Discussion:**

EMBalance is the first trial to test the accuracy of a DSS in both the diagnosis of and the management plan for vestibular disorders across the healthcare systems of four different countries. The EMBalance study is the result of a combined effort of engineers and physicians to develop an accurate tool to support non-specialist doctors, with no risk for the patient. This trial will provide reliable information about the benefits of implementing DSSs in primary care while supporting the feasibility of testing the EMBalance algorithms in further research.

**Trial registration:**

ClinicalTrials.gov NCT02704819. Registered 29 February 2016.

**Electronic supplementary material:**

The online version of this article (doi:10.1186/s13063-016-1568-x) contains supplementary material, which is available to authorized users.

## Background

Human balance is crucial for an individual’s mobility and independence. Balance depends on integration of vestibular, vision and proprioceptive sensory input within the central nervous system and generation of appropriate motor and other responses that support stance, locomotion and orientation [1]. Dizziness and imbalance symptoms are amongst the most common reasons for visits to a doctor and affect up to 30–40 % of the population by 60 years of age [2, 3]. Healthcare service provision to address vestibular disorders remains inadequate and is regarded as a low priority [4]. Non-specialist physicians may feel overwhelmed when faced with a patient complaining of ‘dizziness’, owing to the vagueness of this symptom and the plethora of underlying pathologies. Narrowing the symptom to the causative system (e.g., cardiovascular, neurological, otological) or as being iatrogenic (e.g., a drug-related adverse reaction) is a challenging process. Unsurprisingly, in both the United States and the United Kingdom, patients require an average of 4.5 visits with their healthcare providers before receiving a correct diagnosis and appropriate treatment plan [4].

The complexity of balance control mechanisms, the lack of medical expertise, and the absence of specialised equipment can be contributory factors to this late diagnosis and the resulting mismanagement of patients with balance disorders [5, 6]. This has a considerable socio-economic impact on the affected individual and the individual’s family and exerts a high burden on society and the health services [7]. Misdiagnosis and mismanagement lead to increased personal burden on the patient and a wide-scale burden on health economics and society [8].

Advances in computer science and artificial intelligence have allowed the development of computer systems that support clinical diagnosis or therapeutic and treatment decisions based on individualised patient data [9, 10]. Decision support systems (DSSs) in particular are aimed at codifying and strategically managing biomedical knowledge to handle clinical challenges using mathematical modelling tools, medical data-processing techniques, and artificial intelligence methods [11–13]. However, a review of existing DSSs used in medicine demonstrated that there are but few successful integrated software systems or stand-alone tools which address the early diagnosis and effective management of balance disorders [14]. Current DSSs mainly target diagnosis, but none provide management and/or rehabilitation support. There is also limited information in the time domain (follow-up visits) and very limited literature on detailed balance-related models, while there are no vestibular models currently integrated into a DSS or used in clinical practice, and no patient-specific models [14].

EMBalance is a European project funded by the European Union’s Seventh Framework Programme for Research. Its aim is to develop a DSS to support clinical decision-making, facilitate accurate diagnosis and advise on efficient treatment of balance disorders. The final outcome of the EMBalance project will be an Internet-based platform provided to primary and secondary care physicians across specialties, all levels of training, and with no geographical boundaries, with the objectives of early diagnostic evaluation and effective management of balance disorders.

### The EMBalance decision support system

The EMBalance DSS is a multilanguage platform that consists of three modules: the database, the back end and the graphical user interface (GUI):

- The database of the system is the implementation of the repository. The repository has been modelled using entity relationship modelling notation and MySQL Workbench, an open source software programme that has been developed by Oracle (Redwood Shores, CA, USA) and supports a relational database-modelling and development approach. MySQL Workbench was also used for the administration of the repository. In total, the EMBalance repository is composed of 48 entities, including instance tables (actual clinical data collected from 1100 patients’ records), and type tables, which are the values used to populate an attribute of the instance tables (e.g., patient occupation). Of these, 16 tables store many-to-many relationships.
- The back end is used to implement the functionalities of the system and is composed of the configuration, the entities, the services and the repository.
- The GUI, also known as *front end*, is a user-friendly and easy-to-understand Internet-based tool that the clinician uses to input patient information, which subsequently feeds into the repository to generate assisted diagnosis and management outcomes. The interface has been developed using the Spring Framework for the back end of the tool and the Ext JS Framework for the GUI.

The primary users of the platform are non-specialist doctors (e.g., general practitioners), and its intended use has the following characteristics:

- The doctors can pace the process any way they see fit (e.g., by switching from history-taking to examination, stopping at any point or going back to medical history) or by stopping the process entirely.
- The doctors who use the DSS will be asked to exercise their clinical judgement in order to come up with a diagnosis or management plan.
- Although the EMBalance DSS platform will propose two or three diagnoses (with probability estimation for each), the doctors will be asked to either choose one of these or discard them and choose their own.
- At the end of this process, a specialist consultant will evaluate the patient to provide the definitive patient diagnosis and management plan; hence, patient care will not be affected other than by a longer-winded process for the patient.

## Methods/design

EMBalance is a feasibility and/or proof-of-concept study. It is designed as a non-commercial, international, multi-centre, single-blind, parallel-group randomised controlled trial (RCT) in accordance with the Standard Protocol Items: Recommendations for Interventional Trials (SPIRIT) guidelines [15] (see Additional file 1). The EMBalance study will be carried out simultaneously in the United Kingdom, Germany, Greece and Belgium. Both primary and tertiary care settings will be involved in these four countries. Table 1 provides the names of all participating clinical centres.

**Table 1:** Participating centres: a comprehensive list of primary and tertiary care settings in the EMBalance study across Europe

Institution	Primary care setting	Tertiary care setting
Greece (University of Athens)	Hippocrateio Hospital	Hippocrateio Hospital
Belgium (Antwerp University)	Antwerp University Hospital	Antwerp University Hospital
Germany (University of Freiburg)	Freiburg University Medical Centre	Freiburg University Medical Centre
UK (University College London)	Keats Group Practice	National Hospital for Neurology and Neurosurgery
	Hampstead Group Practice	
	Parliament Hill Practice	
	James Wigg Practice	
	Brondesbury Medical Centre	

### Study participants and subject selection

The primary participants in this study are the non-specialist doctors as users of the DSS. This is to provide a proof of concept of the DSS in aiding diagnosis and management of the ‘dizzy’ patient. Participating doctors are divided into two groups: non-specialist doctors and overseeing experts.

#### Non-specialist doctors

The non-specialist doctors are defined as those who do not necessarily focus their clinical interest in balance disorders and who manage a range of patient categories. These doctors are the first point of access for patients seeking medical advice. All participating non-specialist doctors will be registered with their local medical boards, will have completed all required basic medical or other allied health education, and will have some clinical experience in managing patients in general. All non-specialist doctors will receive a 1-h training session during the site induction clinic on how to use the EMBalance DSS platform. The group of non-specialist doctors in each country will be composed of the following:

- UK: general practitioners
- Belgium and Greece: ear, nose and throat (ENT) residents
- Germany: neurology residents

#### Overseeing experts

The overseeing experts will be members of the EMBalance consortium, who are highly experienced in the management of vestibular disorders. They will verify the appropriate diagnosis and management of each patient whilst being blinded to the previous use of the DSS by the non-specialist doctor. They will represent the gold standard for vestibular diagnosis and management. The expertise of the consortium is well documented, based on academic profile, years of experience, number of publications in relevant journals and conferences as well as relevant chapters in textbooks, and leading roles in educational and professional associations. The group of overseeing experts in each country is composed of the following:

- United Kingdom: consultants in audiovestibular medicine (AVM)
- Belgium and Greece: ENT specialists with >10 years’ expertise in AVM/neuro-otology
- Germany: neurologists

Patients will be recruited into the study on the basis of the inclusion and exclusion criteria listed in Table 2.

**Table 2:** Inclusion and exclusion criteria

Inclusion criteria
• Aged 18–90 years • Capable of understanding the information provided • Absence of dementia and/or uncontrolled psychiatric disorder • Vertigo or chronic dizziness exacerbated by head movements (<12 months) • Sub-acute presentation of dizziness (0–3 months) without presenting to emergency services
Exclusion criteria
• Aged <18 or >90 years • Learning disabilities or dementia • Uncontrolled psychiatric disorders • Pregnant and breastfeeding women • Incapable of or unwilling to give informed consent • Acute vestibular disorders (<3 months) presented at accident and emergency services

Patients who present with balance-related symptoms (specifically, vertigo or dizziness exacerbated by head movements) in primary care will be randomised into one of two groups:

- *Intervention group* (non-specialist doctor + DSS): Patients will be seen by a non-specialist doctor with the support of the DSS.
- *Control group* (non-specialist doctor − DSS): Patients will receive a standard visit with the non-specialist doctor without use of the DSS.

Ultimately, all patients’ cases will be reviewed by an overseeing expert, who will validate the clinical information collected. The overseeing expert will rate the diagnosis and management decisions made by the non-specialist doctor (both with and without the DSS) as correct or incorrect to assess whether the use of the EMBalance DSS leads to the correct diagnosis and improved management. In the event of disagreement, the overseeing expert will provide the final diagnosis and management plan for the patient according to evidence-based guidelines.

### Schedule of intervention

The EMBalance feasibility study is comprised of two parts: part I, in which patients are reviewed by the non-specialist doctors with or without the DSS to come to a diagnosis and devise a care plan, which is then reviewed by the overseeing expert; and part II, which refers to the management arm of the study and follow-up of all patients included. Part II will be carried out in the United Kingdom and Greece exclusively. A study diagram summarising the recruitment and intervention procedures is provided in Additional file 2.

#### Internal pilot

Owing to the limited time frame, this project includes an internal pilot phase. The first two non-specialist doctors and four to six patients entering the trial will provide a realistic overview of the potential barriers in connection with this protocol and study design. This is expected to happen during the first month of the trial. The pilot phase will follow the same study procedures described for parts I and II.

#### Part I: diagnosis and management plan assessment

On the day of recruitment, patients will initially be examined by the non-specialist doctor, either with or without the DSS, according to randomisation result. In both the + DSS and − DSS groups, each patient will undergo the investigations required, following which the non-specialist doctor will formulate an initial diagnosis and management plan. The patient will then be invited to attend a specialist neuro-otology clinic, where they will be seen by an overseeing expert to undergo a gold standard diagnostic process and to determine the management plan appropriate to the diagnosis, which will be compared with the management plan previously advised by the non-specialist doctor. The overseeing expert will review investigations carried out and results assessed by the non-specialist doctor. In the event that the doctor’s assessment differs from the conclusion of the expert, the latter will decide on the final diagnosis and management plan of the patient according to current evidence-based guidelines.

#### Part II: delivery of the vestibular rehabilitation programme

Those patients who require vestibular physiotherapy will enter the second part of the study. Alternatively, for patients not eligible for vestibular physiotherapy, the DSS will propose an alternative management plan, such as pharmacological treatment or a dietary intervention. Independently of the management plan applied, all patients will be reviewed after 3-month follow-up by the overseeing expert.

Once the diagnosis and treatment plan are agreed, patients requiring the vestibular rehabilitation programme will receive a 3-month treatment as explained below:

- *+ DSS group*: Patients will be seen by a non-specialist physiotherapist on a monthly basis over a 3-month period. This treatment will consist of customised vestibular exercises. Such exercises are based on the eye, head and postural exercises that provoke a patient’s symptoms. Exercises incorporating gaze fixation, head movements and postural exercises are prescribed to promote adaptation of vestibulo-ocular reflex and vestibulospinal reflex function. The patient will practice up to five exercises at home for approximately 1–2 minutes each, twice daily, initially at a slow speed and gradually increasing as symptoms improve. Patients presenting with vestibular migraine will perform a maximum of three exercises. These exercises will be chosen by the DSS from among a range of established exercises.
*− DSS group*: Participants will receive routine vestibular physiotherapy as per standard current practice. The standard rehabilitation programme might include lifestyle advice and education, sometimes accompanied by a leaflet.

#### Follow-up

All participants will be reviewed after 3 months from their inclusion in the trial for a follow-up appointment with the specialist doctor.

### Criteria for discontinuation and end-of-study definition

Participants can withdraw from the trial at any time or if, in the opinion of the investigator or clinical team, it is medically necessary to do so. Reasons for withdrawal will be documented; participants who withdraw will be returned to routine care, and no further data will be collected. In the case of adverse events, these will be recorded in the medical records, and an adverse event form will be completed and emailed to the sponsor within the next 5 working days. When the last enrolled participant has completed the follow-up, the research ethics committee (REC) will be notified of the trial completion. The final study report will be completed within 12 months after trial completion.

### Trial objectives

#### Primary objectives

The primary objectives of this study are (1) to determine whether the EMBalance DSS is an accurate supportive tool for the diagnosis and management plan of dizziness and/or balance disorders when used by non-specialist doctors and (2) to establish the feasibility of testing the DSS in a large-scale RCT. These objectives will be measured as follows:

- Percentage of agreement between the diagnosis and management plan established by the non-specialist doctors (using the DSS and not using the DSS) and the gold standard as determined by an overseeing expert and according to current evidence-based guidelines for the diagnosis and/or management of these disorders

The primary outcome assessment criteria are shown in Table 3.

**Table 3:** Primary outcome assessment criteria

Diagnosis (Dx)				
Single		Concurrent		
If doctorDx = expert Dx	100 %	If doctorDx_1_ + Dx_2_ = expertDx_1_ + Dx_2_		100 %
If doctorDx ≠ expert Dx	0 %	If doctorDx_1_ + Dx_2_ ≠ expertDx_1_ + Dx_2_		0 %
		If doctorDx_1_ = expertDx_1_ and doctorDx_2_ NA/≠ expertDx_2_		50 %
Management (Mng)				
Single		Multiple		
If doctorMng = expertMng	100 %	Maximum number of correct Mng plans = 2	If doctorMng_1,2_ = expertMng_1, 2_	100 %
If doctorMng ≠ expertMng	0 %		If doctorMng_1,2_ ≠ expertMng_1,2_	0 %
			If doctorMng_1_ = expertMng_1_ and doctorMng_2_ NA/≠ expertMng_2_	50 %
		Maximum number of correct Mng plans = 3	If doctorMng_1,2,3_ = expertMng_1,2,3_	100 %
			If doctorMng_1,2,3_ NA/≠ expertMng_1,2,3_	0 %
			If doctorMng_1_ = expertMng_1_ and doctorMng_2,3_ NA/≠ expertMng_2,3_	33.3 %
			If doctorMng_1,2_ = expertMng_1,2_ and doctorMng_3_ NA/≠ expertMng_3_	66.6 %
		Maximum number of correct Mng plans = 4	If doctorMng_1,2,3,4_ = expertMng_1,2,3,4_	100 %
			If doctorMng_1,2,3,4_ ≠ expertMng_1,2,3,4_	0 %
			If doctorMng_1_ = expertMng_1_ and doctorMng_2,3,4_ NA/≠ expertMng_2,3,4_	25 %
			If doctorMng_1,2_ = expertMng_1,2_ and doctorMng_3,4_ NA/≠ expertMng_3,4_	50 %
			If doctorMng_1,2,3_ = expertMng_1,2,3_ and doctorMng_4_ NA/≠ expertMng_4_	75 %
Diagnosis		100 % = correct		
		50 % = half correct		
		0 % = incorrect		
Management		100 % = fully correct		
		>50 % = majority correct		
		50 % = half correct		
		<50 % = partially correct		
		0 % = incorrect		

These criteria determine the level of agreement between the non-specialist doctor and the overseeing expert

#### Secondary objectives

The following secondary objectives are also being evaluated in the trial:

- Assessment of the usability of DSS in primary care
- Determination of the number of referrals to secondary care needed in both the + DSS and − DSS groups
- Determination of the number and cost of investigations required for an accurate diagnosis by the DSS non-specialist doctor user (+DSS) vs. the non-DSS non-specialist doctor user (−DSS)
- Effectiveness of treatment proposed by DSS vs. treatment proposed by non-DSS non-specialist doctor
- Assessment of feasibility factors (participation rate, dropout rate, reason for exclusion from the trial, trial accomplishment with the time frame)

### Sample size

Sample size was not formally calculated, owing to lack of available data with respect to standard deviation. However, 200 participants should provide sufficient power for the analysis. Participants will be equally distributed in the 4 countries; that is, 50 patients will be recruited in each country. Regarding physicians, each country site will collaborate with 2 overseeing experts and 10–15 non-specialist doctors. The number of patients recruited per centre will be determined by the number of non-specialist doctors available at each site. This is because non-specialist doctors are expected to learn from the EMBalance DSS, as the system will guide the doctors through the diagnostic procedures with access to supportive learning material, such as video tutorials on diagnostic manoeuvres. To minimise the contamination effect that this might have on non-specialist doctors’ decisions, each clinician will be expected to see a maximum of six patients.

Competitive enrolment programs will not be implemented. This decision was adopted in accordance with recent evidence which suggests that competitive enrolment practices have not proved a significant effect in study recruitment, while posing ethical dilemmas and compromising the study design [16].

### Recruitment procedures

The recruitment strategy was adjusted to the characteristics of each participating centre. Potential participants will be identified when they contact the research clinic. The staff member who serves as the point of contact will ask the patient whether he or she experiences dizziness or vertigo. A patient who confirms these symptoms will receive a study information leaflet to consider participation.

### Sequence generation

Randomisation sequences have been created for each centre using Research Randomizer v4.0 software [17]. Eligible patients will be randomised in a 1:1 ratio (100 participants in each group). In accordance with the random allocation sequence, a note containing the allocation group will be placed inside an opaque, sealed envelope which will be given to the non-specialist doctor at the time of recruitment.

### Allocation concealment

The allocation sequence will be concealed from the researcher by enrolment of participants via sequentially numbered envelopes. Additionally, a patient identification trial number will be assigned to each envelope, which will permit retrospective monitoring of patient allocation. The research nurse will be instructed to strictly follow this sequence.

### Blinding

The EMBalance trial is a single-blind study. The clinical research nurse responsible for patient recruitment and overseeing experts will be blinded.

### Data collection

Non-specialist doctors and patients will enter the data in bespoke case report forms (CRFs) in paper format, and the CRF data will be transferred into a purpose-built electronic database. CRFs were designed and will be completed with reference to the Joint Unit Guide for the Design, Use and Completion of Paper Case Report Forms. The completion of CRFs will be signed off by the chief investigator (CI) or a delegated authorised individual. A blank hard copy of the CRF was submitted for review by the REC and the sponsor. A copy is also included within the trial master file.

### Statistical analysis

Data analysis will be performed using SPSS version 16.0 software (SPSS, Chicago, IL, USA) and the StatXact statistical software package (Cytel, Cambridge, MA, USA). Statistical significance will be set to 0.05. Descriptive statistics will be calculated and presented. Comparisons which will be made include the following:

- Proportion of correct diagnosis by specialists to non-specialists and treatments with and without DSS use according to the overseeing expert’s diagnosis and treatment
- Comparison of the number of laboratory investigations prescribed with and without DSS use
- Comparison of the number of investigation referrals with and without DSS use
- Comparisons of improvement according to the patient at baseline and after 3 months of follow-up, based on the following tool scores: Dizziness Handicap Inventory, EQ-5D-3 L Questionnaire, visual analogue scale scores before and after treatment in the DSS vs. non-DSS user groups

For categorical values (e.g., referral rate), the chi-square test will be used for comparisons between groups. The Bonferroni correction will be used in cases of multiple comparisons. Normality will be tested with the Kolmogorov-Smirnov test. If normality is met, Student’s *t* test will be used. If not, a non-parametric Mann-Whitney *U* test will be used instead. For comparisons before and after treatment, a paired *t* test will be used regarding numeric data (e.g., questionnaire scores) after normality testing.

Because of the close monitoring of all study participants, the proportion of missing data is not expected to be substantial. In the unlikely event that more than 10 % of values corresponding to primary outcome measures are missing, recruitment will be extended to collect additional data.

### Monitoring and auditing

Adherence to the protocol, procedures for reporting adverse events, and adequate data quality will be supervised by the sponsor. In addition, a trial steering committee composed of independent experts was established for examining legal, ethical and data protection as well as privacy and security issues related to the research activities during the whole project life cycle. The CI will inform the sponsor should she have concerns that arise from monitoring activities and/or if there are problems with oversight and/or monitoring procedures.

### Confidentiality

Patient anonymity is protected and maintained by using a unique identification trial number. No personal data will be collected as part of the study, although sensitive information such as ethnicity and physical condition could be collected. Participants will be informed about the transfer of this information to the study office and will be asked to consent to this. At all research sites, staff share the same duty of care to prevent unauthorised disclosure of personal information. All personal information obtained for the trial will be held securely, treated as strictly confidential and managed in accordance with the Data Protection Act, the NHS Caldicott guardian, the Research Governance Framework for Health and Social Care, and REC approval.

### Archiving

During the course of the study, all records are the responsibility of the CI and will be kept in secure conditions. When the trial is complete, the CI will confirm that the study master file will be archived for 20 years, as stipulated in line with all relevant legal and statutory requirements. For this purpose, the master file will be transferred to an approved long-term repository.

## Discussion

The EMBalance project combines the efforts of experts from the fields of bioengineering and medicine to create a tool to support non-specialist doctors in their decision-making. The EMBalance DSS was developed to aid healthcare professionals in primary care while enabling clinicians to undertake the final decision about the management and treatment of the patient. The design of this protocol has been a challenge for the authors due to the need to conceive of a harmonised protocol suitable for all clinical settings (e.g., composition of a non-specialist doctors group). The present document establishes the key requisites to obtain a unified set of data from all participating countries while taking into account the potential benefits derived from both doctors’ and patients’ participation.

From the patient’s perspective, the use of the EMBalance platform is a minimally invasive intervention which is accompanied by multiple advantages: (1) better and earlier diagnosis and treatment for the balance disorder, (2) review of the participant’s case by a renowned expert and (3) improvement of the patient’s quality of life. Despite the support of the EMBalance DSS, however, non-specialist doctors are asked to exercise their clinical judgement, which ultimately will be compared with an expert’s opinion. Although at first glance this could negatively affect doctors’ willingness to participate in the trial, it will benefit their participation by providing guidance for the diagnostic procedure of the ‘dizzy’ patient, access to supporting learning material, and a unique chance to receive feedback from national and international experts in neuro-otology.

The use of a computer-aided system has been shown to create some disruption of the patient-doctor relationship [9]. The design of the DSS interface, ease in entering patient data and increased appointment time given to consultation are some preliminary measures taken to reduce this limitation and patient discomfort. Furthermore, a special section in the users’ manual is dedicated to informing users regarding this issue and suggesting ways to reduce this risk. The latter includes increasing the efficiency of entering data, keeping within the time constraints of the consultation and maintaining eye contact to promote a good patient-doctor relationship, and further emphasis is placed on the DSS as an auxiliary tool and not a requirement. In the same line, the EMBalance DSS has been reviewed by the Medicines and Healthcare products Regulatory Agency, which determined that, in its current state, the EMBalance DSS does not meet the definition of a medical device defined within Article 1 of Directive 93/42/EEC. This verdict was based on the fact that the EMBalance DSS is a supportive tool and is not intended to be a substitute for the clinician’s decision-making capacity.

The EMBalance trial has been designed to gather, and will be conducted with the ultimate aim of obtaining, further evidence on the development of a DSS that will lead to lower socio-economic costs and foster equality of access to high-quality services for diagnosis and management of vestibular disorders. This proof-of-concept study will determine the feasibility of implementing the EMBalance platform in primary care settings across Europe and will provide a realistic prospect of the accuracy of this innovative tool in addressing the early diagnosis and effective management of balance dysfunctions.

## Trial status

Recruitment started in March 2016, and it is expected to continue until September 2016.

## Additional files

Additional file 1: SPIRIT checklist. (DOC 121 kb) Additional file 2: EMBalance study diagram synthetizing the progress through the phases of the EMBalance study from screening to follow-up. (PDF 151 kb)

## Abbreviations

AVM: Audiovestibular medicine
CI: Chief investigator
CRF: Case report form
DSS: Decision support system
Dx: Diagnosis
ENT: Ear, nose and throat
EU: European Union
GUI: Graphical user interface
Mng: Management
REC: Research ethics committee
RCT: Randomised controlled trial
SPIRIT: Standard Protocol Items: Recommendations for Interventional Trials
UCL: University College London
JRMO: Joint Research Management Office
