# Supplementary figures and images for: EMBalance - validation of a decision support system in the early diagnostic evaluation and management plan formulation of balance disorders in primary care: study protocol of a feasibility randomised controlled trial
Source: Trials. 2016 Sep 5;17(1):435. doi: 10.1186/s13063-016-1568-x (PMC5011840; doi:10.1186/s13063-016-1568-x)

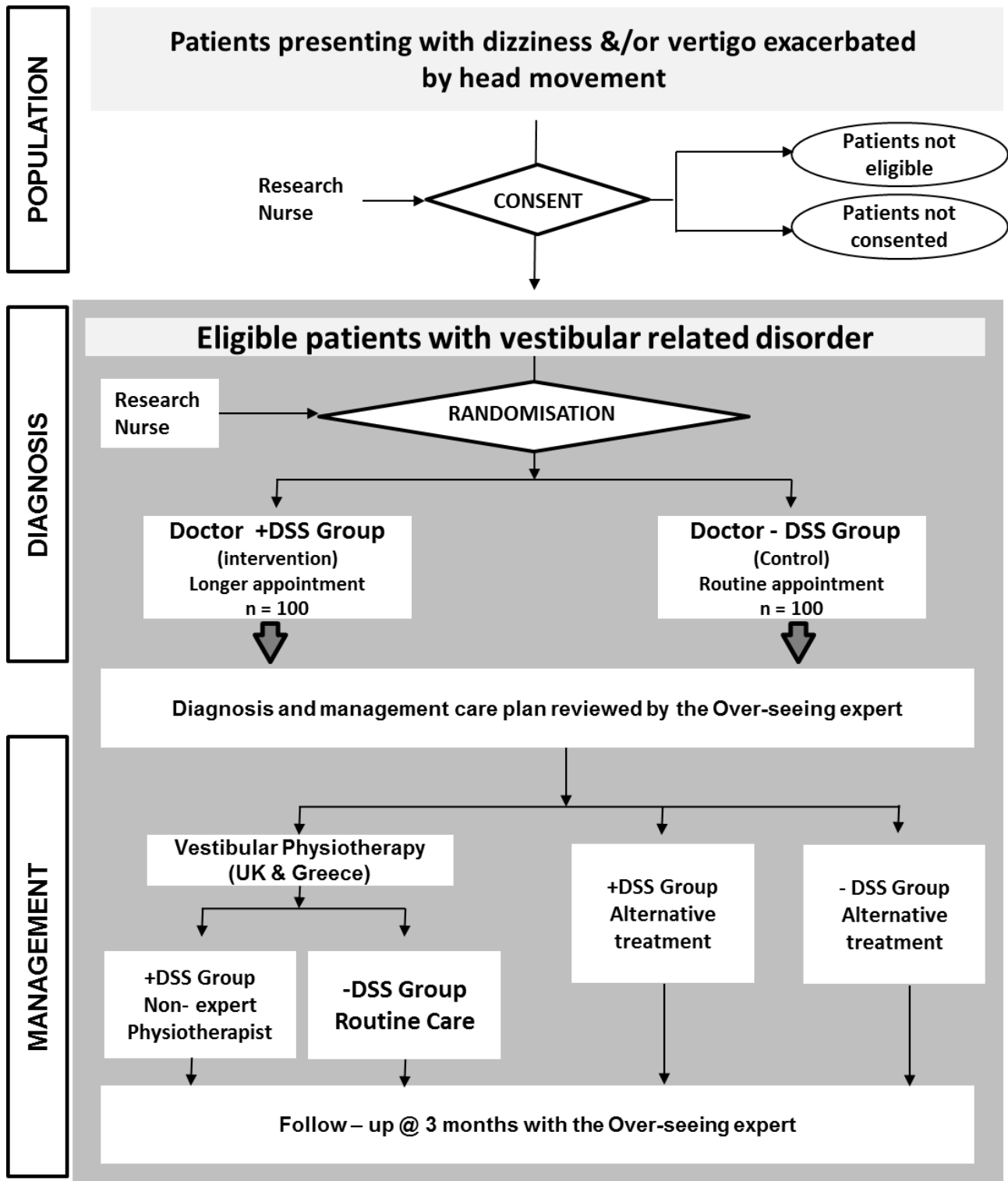

Supplement: Additional file 2: — EMBalance study diagram synthetizing the progress through the phases of the EMBalance study from screening to follow-up. (PDF 151 kb) [file 13063_2016_1568_MOESM2_ESM.pdf]
